# Clinical Outcomes and Patterns of Neurological Toxicity After Stereotactic Body Radiotherapy Reirradiation (reSBRT) of Spine Metastases Previously Treated with SBRT

**DOI:** 10.3390/cancers18081301

**Published:** 2026-04-20

**Authors:** Ahmed N. Elguindy, Eric R. Cochran, Khaled N. Dibs, Katelyn Fernando, Mark Addington, Eugene Yap, Robyn Handschuh, Dominic J. DiCostanzo, Daniel Schneider, Brian Park, James B. Elder, Russell Lonser, Daniel Boulter, Eric C. Bourekas, David J. Konieczkowski, Sasha Beyer, Simeng Zhu, Raj Singh, Raju Raval, John C. Grecula, Arnab Chakravarti, Joshua D. Palmer, Dukagjin M. Blakaj

**Affiliations:** 1 Department of Radiation Oncology, The James Cancer Hospital/Wexner Medical Center, The Ohio State University, Columbus, OH 43210, USA; elgu02@osumc.edu (A.N.E.); eric.cochran@osumc.edu (E.R.C.);; 2 Department of Radiation Oncology, El-Demerdash Hospitals, Ain Shams University, Cairo, Egypt; 3 Department of Neurosurgery, The James Cancer Hospital/Wexner Medical Center, The Ohio State University, Columbus, OH 43210, USA; daniel.schneider@osumc.edu (D.S.);; 4 Department of Radiology, The James Cancer Hospital/Wexner Medical Center, The Ohio State University, Columbus, OH 43210, USA

**Keywords:** SBRT, stereotactic, myelitis, myelopathy, vertebral fracture, reirradiation

## Abstract

Background/Objectives: Stereotactic body radiation therapy (SBRT) provides improved pain response and local control for spinal metastases. However, management of local failure after initial SBRT is challenging. We report institutional outcomes, dosimetry, and toxicity for reSBRT following SBRT. Methods: We retrospectively reviewed 61 lesions (55 patients) treated with reSBRT after prior SBRT. Both SBRT courses delivered a median dose of 27 Gy. Patients underwent clinical and radiological evaluation every three months. Toxicity was graded using CTCAE v5.0. Dosimetric parameters for the spinal cord (SC), cauda equina (CE), planning organ-at-risk volumes (PRV), and thecal sac were converted to equivalent dose in 2 Gy fractions (EQD_2_) using the linear–quadratic model (α/β = 2). Results: Median follow-up was 10.3 months. Forty lesions (65%) were cervicothoracic and 21 (35%) were lumbosacral. One- and two-year overall survival (OS) were 45% and 29%, respectively, and one- and two-year local control (LC) were 89% and 88%, respectively. Gastrointestinal primary tumors were associated with inferior LC (HR 2.41, 95% CI 1.11–5.23, *p* = 0.026). Fifteen patients (27%) reported myelitis/neuropathic symptoms during follow-up; four (7%) developed new post-radiation myelitis or neuropathy (RMN) without radiologic progression. Five patients (9%) developed vertebral compression fractures (VCF). Cumulative EQD_2_ was not significantly associated with RMN (*p* = 0.344); all affected patients had thecal sac EQD_2_ > 95.5 Gy and relevant nerve roots EQD_2_ > 108 Gy. Conclusions: ReSBRT provided a favorable LC with acceptable toxicity. High cumulative dose to the thecal sac and nerve roots may contribute to neurologic toxicity as peripheral nerve injury.

## Simple Summary

Spinal metastases can cause severe pain and neurological problems, and radiation is a common treatment. Stereotactic body radiation therapy is a focused form of radiation that can control spinal metastases better than standard radiation; however, spinal metastases may recur after treatment. Delivering stereotactic reirradiation to the same or nearby spinal area is challenging because the spinal cord and nerves may be exposed to high cumulative radiation doses. In this study, we reviewed patients who received stereotactic reirradiation after a previous stereotactic course and assessed tumor control, survival, side effects, and the total radiation dose received by critical neurological structures. We found high rates of local tumor control with relatively low rates of serious nerve injury. Our results suggest that repeat stereotactic radiation can be effective, and that careful attention to dose to the spinal canal and nerve roots may help reduce neurologic toxicity.

## 1. Introduction

Spinal metastases represent a frequent site of cancer dissemination, with a cumulative incidence of approximately 15% among patients with solid malignancies [1]. Conventional external beam radiation therapy (cEBRT) has been the mainstay of treatment, providing symptomatic relief in up to 66% of cases [2]. However, local failure after cEBRT is not uncommon, with pain control failure rates reaching 40% [3]. Additionally, 30% of patients may experience local progression within a year of treatment with cEBRT, particularly those with radio-resistant histologies or limited target coverage [4].

To address these limitations, spine stereotactic body radiation therapy (SBRT) has emerged as a more precise and effective modality for spinal metastases treatment [5]. By delivering high-dose radiation with steep dose gradients, SBRT improves local tumor control while minimizing dose to the spinal cord (SC) [6].

A recent phase III randomized controlled trial demonstrated significantly improved pain control with SBRT compared to cEBRT, with 3-month complete pain response rates of 35% versus 14%, and sustained benefits at 6 months (32% versus 16%) [7]. Moreover, retrospective analysis revealed 1–2-year lower local failure rates with SBRT (6.1–14.8%) compared to cEBRT (28.4–35.6%), and markedly reduced need for reirradiation (2.2% versus 15.8%) [8].

Historically, reirradiation of the spine has been approached with caution due to the significant risk of SC toxicity, which could result in irreversible radiation-induced myelitis/neuropathy (RMN) [9]. However, more recent data suggest that with appropriate patient selection and dose constraints, reirradiation with SBRT is associated with low rates of SC toxicity [10,11,12].

While reSBRT after cEBRT has been relatively well studied, data are limited on reSBRT following a prior course of SBRT. This study presents one of the largest reported cohorts of patients treated with spine reSBRT after initial SBRT due to local failure.

## 2. Materials and Methods

### 2.1. Study Design

After Institutional Review Board (IRB) approval, we conducted a single-institution retrospective study on patients who underwent reSBRT following local failure after prior SBRT between 2016 and 2024. ReSBRT was primarily offered for radiographic local progression on follow-up imaging. Clinical symptoms such as pain or neurologic deficits were present in a subset of patients; however, these were not uniformly captured in a standardized manner due to the retrospective design.

Reirradiation was defined based on ESTRO consensus as a new course of radiotherapy, either to a previously irradiated volume (e.g., same spine level) or where the cumulative dose raised concerns for toxicity (adjacent spine-level tumor) [13].

Mechanical stability was assessed using the spinal instability neoplastic score (SINS) [14], and the extent of epidural disease was assessed using the epidural spinal cord compression (ESCC) scale, also known as Bilsky grading [15]. Primary endpoints included local control (LC) and radiation-induced myelitis/neuropathy (RMN) after the reSBRT. RMN was analyzed as a patient-level endpoint, consistent with CTCAE grading. Additional secondary endpoints included overall survival (OS) and vertebral compression fracture (VCF).

### 2.2. Imaging and Simulation

Planning CT simulations were performed, typically without contrast, with 1–2 mm axial slices covering the involved spinal segment and adjacent levels (e.g., including cervical and thoracic spine for C7 or T1 lesions). Immobilization was achieved using custom vacuum immobilization devices and stereotactic body frames, with thermoplastic head and neck mask–based immobilization for upper thoracic (T5 and above) lesions. MRI of the spine was used for image co-registration to delineate the tumor, SC, and cauda equina (CE). Although 3-T MRI was usually used, 1.5-T MRI was employed in select postoperative cases with spinal instrumentation, including carbon-PEEK hardware, based on institutional experience and artifact considerations [16]. MRI acquisitions followed standard clinical spine protocols and included high–spatial resolution sequences (typically 1–2 mm slice thickness), comprising T1-weighted, T2-weighted, and post-contrast T1-weighted imaging for target and neural structure delineation.

### 2.3. Volumes Delineation and Treatment Planning

Treatment planning was performed using the Eclipse Treatment Planning System (version 18.1, Varian Medical Systems, Palo Alto, CA, USA), and clinical workflow and data management were conducted using the ARIA Oncology Information System (version 18.0, Varian Medical Systems, Palo Alto, CA, USA).

The gross target volume (GTV) was contoured on the T1 post-contrast MRI, including the epidural extension if present. The clinical target volume (CTV) was defined following international consensus guidelines for intact, sacral, and postoperative spine SBRT, respectively [17,18,19].

The planning target volume (PTV) was set equal to the CTV (i.e., no additional margin). A PTV_Eval structure was generated by copying the PTV and cropping it away from SC and CE planning organ-at-risk volumes (PRV), prioritizing OAR sparing over PTV coverage. It was acceptable to incorporate adjacent contiguous vertebral levels invaded by the same tumor into a single PTV (e.g., PTV_T1-3).

The SC and CE were contoured based on T2-weighted axial and/or sagittal Sampling Perfection with Application-optimized Contrasts using different flip angle Evolutions (SPACE) Short Tau Inversion Recovery (STIR) MRI sequences. The SC/CE contours were extended at least one vertebral body level above and below the target PTV to capture dosimetric effects from non-coplanar beams. A planning risk volume (PRV) was added as an additional 2 mm expansion to account for setup uncertainty and physiological SC motion in CSF [20,21]. Lumbosacral plexus and nerve roots outside the spinal canal were contoured as separate OARs.

Although the thecal sac is not a conventional OAR, it was analyzed as a composite neural surrogate to account for overlapping contributions from the SC, conus medullaris, CE, and traversing nerve roots. The thecal sac was contoured on simulation CT as a surrogate for the spinal canal, with MRI co-registration used for anatomic confirmation when available. Reporting cumulative EQD_2_ to the thecal sac enabled standardized dosimetric assessment across spinal levels and facilitated correlation with observed patterns of RMN. All cases underwent peer review. Plans were normalized to cover 95% of the PTV with 100% of the prescription dose. Hotspots were allowed only within the PTV (preferably within the GTV) and were not permitted to overlap true SC or CE volumes.

### 2.4. Plan Evaluation

Cumulative EQD_2_ values were calculated using the linear–quadratic model (α/β = 10 for tumor, α/β = 2 for SC, SC PRV, CE, and CE PRV). Initially, the aim was to keep cumulative EQD_2_ to PRV < 70 Gy. If that aim was not feasible, priority was given to maintain cumulative EQD_2_ to true SC or CE volumes < 70 Gy_2_. Risk stratification was applied based on cumulative Dmax EQD_2_ to the PRV using tables/methods from previous studies [9,22,23]:

- Low risk: EQD_2_ < 75 Gy.
- Intermediate risk: EQD_2_ < 90 Gy (acceptable in selected clinical scenarios).
- High risk: EQD_2_ ≥ 90 Gy (non-acceptable).

### 2.5. Treatment Delivery and Post-Treatment Evaluation

Image guidance included daily orthogonal kV imaging and cone beam CT (CBCT). Clinical assessment and MRI were performed every 3 months for the first 2 years, then every 4–6 months. Response was assessed independently by two neuroradiologists using SPINO (Spine response assessment in Neuro-Oncology) criteria [24]. Radiation toxicity was graded based on the Common Terminology Criteria for Adverse Events (CTCAE) version 5.0 [25]. Radiation myelitis was defined as new or worsening neurologic deficits corresponding to the irradiated spinal segment from C1 to L1, without evidence of recurrent tumor abutting the SC. CE neuropathy was defined as new or worsening neurologic deficits corresponding to the irradiated spinal segment from L2 to S3. Peripheral neuropathy was defined as new or worsening neurologic deficits corresponding to the irradiated nerve roots or peripheral nerves outside the thecal sac at any spinal segment.

### 2.6. Statistical Analysis

Descriptive statistics were used to summarize baseline patient demographics, treatment characteristics, and toxicity profiles. Categorical variables were reported as frequencies and percentages, while continuous variables were summarized using medians and ranges/interquartile ranges.

Time-to-event endpoints were calculated from the date of reSBRT to the date of event (local failure or death) or last follow-up. LC was defined as no radiographic progression within the target volume treated. Radiographic progression was determined based on SPINO criteria on serial MRI evaluation and multidisciplinary review. OS was assessed from the reSBRT course till death or last follow-up. Cases were divided into cervicothoracic and lumbosacral to correlate with SC and CE constraints, respectively. Thoracolumbar junction cases (T12–L2) were grouped with the cervicothoracic cohort for constraint reporting; however, in this anatomic region, attribution of neurologic toxicity accounted for overlap of the treatment volume with the conus medullaris, cauda equina, and traversing nerve roots, as well as the clinical pattern of neurologic deficits. In cases with overlapping anatomic exposure and non-localizing clinical features, toxicity classification was based on the predominant anatomic level and symptom distribution, while dosimetric analyses were reported on a structure-specific basis. LC and OS were estimated using the Kaplan–Meier method, and comparisons between groups were conducted using the log-rank test. Cox regression analysis was conducted to correlate multiple variables with the outcomes. Given the limited number of local failure events, multivariable modeling was intentionally restricted to avoid overfitting and preserve model stability. Statistical analyses were performed using IBM SPSS Statistics for Windows, Version 29.0 (IBM Corp, Armonk, NY, USA).

## 3. Results

### 3.1. Patient Characteristics

We included 61 spinal lesions in 55 patients, with median follow-up of 10.3 months (range: 1–98). The median age was 65 years (range: 27.1–87.3), with 38 males (67%) and 17 females (33%). Karnofsky Performance Status (KPS) was ≥80% in 89% of the cohort. The median interval between initial primary cancer diagnosis and spinal metastases was 12.8 months (range: 0–200).

The most frequent primary malignancy was lung cancer (25%), followed by renal cell carcinoma (18%), gastrointestinal (GI: 15%), thyroid (10%), sarcoma (10%), and prostate cancer (7%). Nine patients (16%) had oligometastatic disease (≤5 lesions). Baseline patient and disease characteristics are detailed in Table 1.

Regarding lesion distribution, 40 (65%) lesions were cervicothoracic and 21 (35%) were lumbosacral. Based on Bilsky grading at initial SBRT, 37 lesions (61%) were low grade (0–1a) and 24 (39%) were higher grade (1b–3). Based on SINS at initial SBRT, 33 lesions (54%) were classified as stable, 27 (44%) as indeterminate, and one (2%) as unstable. Surgical intervention was performed for 23 lesions (38%), primarily vertebrectomy (18%) and laminectomy (16%).

The median interval between SBRT and reSBRT was 10.6 months (range: 0.57–53.9). The reSBRT was delivered to the same vertebral level in 31 cases (51%), while it targeted an adjacent level in 30 (49%) (where overlap or cumulative toxicity concerns applied). Six patients (11%) received the same spine-level reSBRT within a ≤5-month interval due to progression requiring surgical intervention and reirradiation. For the whole group, the first SBRT course delivered a median dose of 27 Gy (range: 24–35 Gy), corresponding to a BED_10_ of 51.3 Gy (range: 37.5–59.5) and EQD_2_ of 42.8 Gy (range: 31.3–49.6). For the reSBRT course, the median dose was 27 Gy (range: 18–30 Gy), with BED_10_ of 51.3 Gy (range: 28.8–51.3) and EQD_2_ of 42.8 Gy (range: 24–42.8). The cumulative BED_10_ was 102.6 Gy (range: 75.0–110.8), corresponding to a cumulative EQD_2_ of 85.5 Gy (range: 62.5–92.3).

### 3.2. Overall Survival and Local Control Outcomes

**Figure 1:**
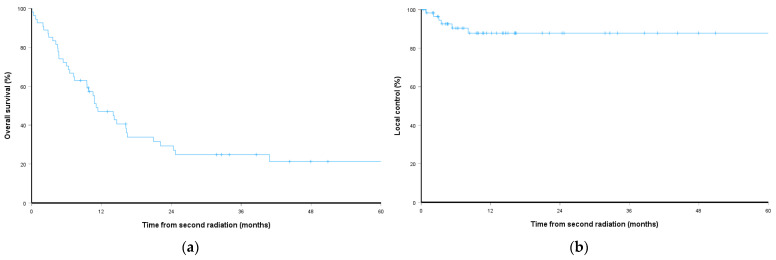
Kaplan–Meier plots: (**a**) overall survival and (**b**) local control.

The median OS after reSBRT was 11.0 months (range: 1–98). The 1-year and 2-year OS rates were 45% and 29%, respectively, as shown in Figure 1. GI origin spine metastases were associated with significantly worse OS (log-rank *p* = 0.014). A longer interval between the initial SBRT and reSBRT was associated with better OS (hazard ratio [HR] 0.95, 95% confidence interval [CI] 0.918–0.984, *p* = 0.005). Variables including oligometastatic disease (*p* = 0.906), Bilsky grading (*p* = 0.247), SINS (*p* = 0.173), prior spine surgery (*p* = 0.256), cumulative EQD_2_ (*p* = 0.157), systemic therapy (*p* = 0.12), and immunotherapy (*p* = 0.302) were not statistically significantly associated with OS.

**Figure 2:**
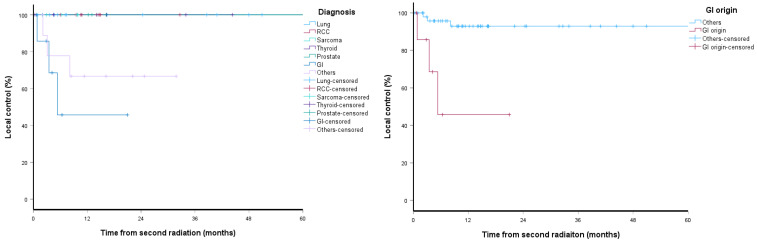
Kaplan–Meier plots of LC between GI and non-GI primary subgroups.

One- and two-year LC after reSBRT were 89% and 88%, respectively, as shown in Figure 1, and the median time to local failure was not reached. In subgroup analysis, LC was significantly worse for GI origin spine metastases compared with non-GI origin spine metastases (log-rank *p* < 0.001), with an estimated 2-year LC of 46% versus 93%, respectively, as shown in Figure 2. On univariate analysis, GI origin spine metastases predicted inferior LC (HR 2.41, 95% CI 1.11–5.23, *p* = 0.026). Age (HR 0.96, 95% CI 0.91–1.02, *p* = 0.164), sex (HR 2.45, 95% CI 0.49–12.16, *p* = 0.273), KPS (HR 1.10, 95% CI 0.98–1.22, *p* = 0.113), Bilsky grading (HR 4.03, 95% CI 0.47–34.51, *p* = 0.204), SINS (HR 1.29, 95% CI 0.31–5.40, *p* = 0.730), spine surgery (HR 0.86, 95% CI 0.16–4.73, *p* = 0.867), cumulative EQD_2_ (HR 0.95, 95% CI 0.85–1.06, *p* = 0.351), and longer interval between SBRT courses (HR 0.88, 95% CI 0.75–1.02, *p* = 0.099) were not statistically significant. In multivariate analysis, GI origin spine metastases remained an independent risk factor for local failure (HR 3.66, 95% CI 1.06–12.64, *p* = 0.041), as shown in Table 2.

### 3.3. Radiation Myelitis/Neuropathy

Fifteen patients (27%) reported myelitis/neuropathic symptoms; nine (16%) had symptoms prior to reSBRT, and four (7%) developed post-radiation symptoms with no radiological evidence of progression. RMN incidence was calculated on a per-patient basis (*n* = 55). Clinical details of patients who developed RMN are summarized in Table 3. Three patients developed radiation myelitis, and one developed peripheral neuropathy. One patient with myelitis received circumferential volume for reSBRT. Another patient with reSBRT involving the thoracolumbar junction (T12–L2) developed a G2 left foot drop with overlapping dosimetric exposure to the conus/cauda and adjacent nerve roots; toxicity attribution favored peripheral neuropathy based on clinical presentation. No significant association was observed between cumulative EQD_2_ and the development of RMN (*p* = 0.344). MRI findings of RMN were observed in two patients in the form of abnormal predominantly central cord signal abnormality within the SC, and a new asymmetric enhancement within the right L4 nerve roots, respectively.

### 3.4. Radiation Details

For the non-RMN subgroup, as shown in Table 4, the median cumulative EQD_2_ Dmax for SC was 27.5 Gy (range: 12.7–69.1), and 54.8 (range: 32.1–121.6) for PRV. The median cumulative EQD_2_ Dmax for CE and PRV were 60.6 Gy (range: 45.6–101.8), and 86.5 Gy (range: 53.5–139.6), respectively. The thecal sac cumulative EQD_2_ Dmax was 73.8 Gy (range: 33.8–148.5) at SC level (above L1), and 92.7 Gy (range: 45.6–199.5) at CE level (L2-S3).

In the RMN subgroup (three patients with reSBRT at/above L1), detailed cumulative dosimetric parameters are summarized in Table 5. The median cumulative EQD_2_ Dmax to SC was 72.5 Gy (range: 33–73.6), and its PRV was 102.4 Gy (range: 86.3–108.7). The thecal sac median cumulative EQD_2_ Dmax was 112.8 Gy (range: 95.5–142.5).

In the CE/peripheral neuropathy subgroup (2 patients with reSBRT at/below L2), the median cumulative EQD_2_ Dmax for CE and its PRV were 65.2 Gy (range: 58–72.5), and 86.4 Gy (range: 86.2–86.6), respectively. The thecal sac median cumulative EQD_2_ Dmax was 107 Gy (range: 95.5–118.5). For all RMN patients, the nerve root median cumulative EQD_2_ Dmax was 148.8 Gy (range: 108.2–179.9).

### 3.5. Non-Neurological Toxicity

Sixteen patients (29%) experienced grade 1–2 fatigue. Twelve patients (22%) had pain flares (managed with short-course steroids). Five patients (9%) developed VCF after reSBRT; three (5.5%) required surgical stabilization for progressive fracture.

## 4. Discussion

Although data on spine reSBRT following an initial SBRT course remain limited, the body of evidence is expanding, and reSBRT has increasingly been adopted given reported favorable LC and acceptable toxicity. In this retrospective study, reSBRT achieved durable LC, with 89% of lesions remaining controlled at 1 year. Although symptom response was not systematically captured, durable local control in the spine is clinically meaningful in preventing neurologic deterioration and maintaining functional status in this population. Median OS was 11 months, which is consistent with the advanced disease stage of most patients (only 16% had oligometastatic disease). Specifically, GI origin spine metastases were a negative prognostic factor for OS on Kaplan–Meier analysis and for LC on univariate and multivariate Cox regression. Treatment was generally well tolerated, with 7% developing RMN.

Few studies have reported reSBRT after a prior SBRT course, similar to our cohort. Thibault et al. reported outcomes of reSBRT in 40 patients (56 lesions) who were treated with a median dose of 30 Gy in four fractions (range: 20–35 Gy in 2–5 fractions), with 32% of them having oligometastatic disease. The median OS was 10 months, and 1-year OS was 48% [10]. Recently, Moore et al. retrospectively analyzed 105 lesions in 102 patients retreated with 40 Gy in five fractions. The median OS was 26 months, with 55% of patients having oligometastatic disease [11]. These differences in patient selection and treatment regimen may explain why our median OS was 11 months and 1-year OS was 45%, more similar to Thibault et al.’s study than to Moore et al.’s study, while long-term LC (89% at 1 year) remained favorable.

In our study, the OS was significantly worse with GI origin spine metastases (log-rank *p* = 0.014), which highlights their relative overall aggressive clinical course. Similar results were also seen with Sandhu et al., who reported a 1-year local failure of 24% for GI-origin spine metastases after stereotactic radiosurgery [26]. These findings suggest that reSBRT outcomes depend on dosimetric delivery and tumor radiobiology and that these patients may need alternative dosing strategies to improve control, such as dose escalation, additional surgery, or systemic therapies.

Additionally, the long interval between SBRT courses correlated with improved OS (HR 0.95, 95% CI 0.91–0.98, *p* = 0.005). Similarly, Thibault et al. observed that patients with a shorter interval between initial SBRT and reSBRT had worse OS (*p* = 0.02) [10]. In contrast, Moore et al. did not find a significant correlation with time from the completion of the first course of SBRT to reSBRT (*p* = 0.19) [11]. This difference could be explained by other variables that may affect OS, such as the primary cancer type, size and number of metastatic lesions, and receiving systemic treatment, which may have been different between the three studies. Additionally, in this study, there was no statistical significance between OS and Bilsky grading (*p* = 0.247), or SINS scoring (*p* = 0.173), or spine surgery (*p* = 0.256). Similarly, systemic therapy and immunotherapy were not significantly associated with OS (*p* = 0.12 and *p* = 0.302, respectively). However, the heterogeneity in timing and type of therapy, along with the limited number of toxicity events, precluded meaningful evaluation of potential interactions with radiation-related neurologic toxicity.

Several earlier series reported reirradiation with SRS/SBRT after previous cEBRT. Gerszten et al. reported outcomes of CyberKnife SRS reirradiation (mean dose 20 Gy in one fraction) after cEBRT in 344 patients with overall long-term radiographic tumor control of 88% [27]. Choi et al. also reported SBRT reirradiation with a median 20 Gy in two fractions for 51 lesions in 42 patients with a 1-year LC rate of 73% [28]. The prospective MD Anderson study by Garg and colleagues reviewed 59 patients with 63 spine lesions and showed actuarial 1-year LC of 76% [29]. Additionally, Hashmi et al. reported a multi-institutional outcome analysis of 215 patients with 247 spine lesions who were treated with SBRT reirradiation to the spine as salvage therapy, with a 1-year LC of 83% [30]. Compared to these studies, our results had better 1-year LC (89%) when patients were treated with SBRT followed by reSBRT upon local progression.

In subgroup analyses, LC was significantly worse for GI primaries compared with non-GI primaries (log-rank *p* < 0.001), with an estimated 2-year LC of 46% versus 93%, respectively. On univariate Cox regression, GI origin was the only variable associated with poorer LC (*p* = 0.026), whereas age, KPS, Bilsky grade, SINS, prior spine surgery, and cumulative EQD_2_ were not statistically significant. Other variables with borderline significance in the univariate analysis were not included in multivariable modeling due to the limited number of events to avoid model overfitting. Similarly, Moore et al. found no association between LC and surgery (*p* = 0.43) or extent of epidural disease (*p* = 0.81) [11].

We also observed a low rate of RMN (4/55: 7%), with a median cumulative EQD_2_ Dmax for the SC of 72.5 Gy (range: 33–73.6), and for the PRV of 102.4 Gy (range: 86.3–108.7). For the CE, the corresponding values for Dmax were 65.2 Gy (range: 58–72.5 Gy) and 86.4 Gy (range: 86.2–86.6 Gy) for the PRV. RMN was analyzed as a composite endpoint given overlapping clinical presentations and limitations of retrospective attribution. However, there was no significant association observed between RMN rates and cumulative EQD_2_ (*p* = 0.344), likely reflecting the limited number of toxicity events. Given the anatomic proximity of spinal nerve roots to the thecal sac and their frequent inclusion within high-dose gradients during reSBRT, distinguishing SC injury from peripheral nerve injury remains challenging.

Similarly, Moore et al. also reported 7.8% RMN (seven neuropathy cases and one myelitis case). However, they reported median cumulative EQD_2_ Dmax of 66 and 90 Gy for the SC and CE, respectively [11]. Thibault et al. reported no radiation myelitis after reSBRT with EQD_2_ Dmax of 74 Gy to the SC PRV; however, this constraint is much less than the SC PRV EQD_2_ Dmax in our RMN cohort (102.4 Gy) [10]. Interestingly, in this cohort, all RMN patients had a cumulative EQD_2_ Dmax > 95 Gy to the thecal sac, and the nerve root median cumulative EQD_2_ Dmax was 148.8 Gy (range: 108.2–179.9). Both values are considerably high compared to TG-101 constraints [22]. The clinical and anatomic pattern of toxicity in our cohort may, in selected cases, reflect peripheral nerve or nerve root injury rather than classic radiation-induced myelitis; however, this distinction remains uncertain and should be considered hypothesis-generating given the limitations of retrospective clinical characterization. Importantly, unlike the SC and major nerve plexuses, there are currently no standardized contouring guidelines or dose constraints for individual spinal nerve roots in spine SBRT or reSBRT. As a result, peripheral nerve injury may be misclassified as radiation myelitis in retrospective series, particularly when symptoms overlap and nerve roots are not routinely delineated.

Regarding non-neurological toxicity, five patients (9%) suffered from VCF after reSBRT, with three of them (5.5%) requiring stabilization surgery, all of whom had previous surgery. Similarly, Moore et al. also reported 16.7% VCF after reSBRT, with no correlation with different variables [11]. However, Thibault et al. reported no VCF after the relatively short follow-up of 6.8 months [10].

This study represents one of the largest retrospective analyses of spine reSBRT after a prior SBRT course, reporting patient outcomes and toxicities. A major strength is the reporting of the dosimetric details of SC, CE, thecal sac, and nerve roots and their association with RMN.

ReSBRT should also be considered within the broader multidisciplinary management of spinal metastases. For example, percutaneous techniques such as radiofrequency ablation combined with vertebroplasty represent complementary approaches for pain palliation and mechanical stabilization, particularly in patients who are not candidates for further radiation due to cumulative dose constraints [31].

However, several limitations should be acknowledged. First, this is a retrospective single-institute study, with risk of selection bias. Additionally, neurologic status at the time of reSBRT was not uniformly documented using standardized scales, which limits detailed clinical characterization of treatment indications. Second, the relatively short median follow-up of 10.3 months may underestimate late-onset neurologic toxicity, including delayed radiation myelopathy, which can occur beyond one year after treatment. Nevertheless, the lack of standardized nerve root contouring further limits definitive differentiation between SC and peripheral nerve injury.

## 5. Conclusions

To our knowledge, this is one of the largest studies to report outcomes of stereotactic radiotherapy (SBRT) followed by reSBRT for spinal metastases after local progression, using 27 Gy in three fractions or 30 Gy in five fractions. Our results showed that reSBRT was associated with a low rate of RMN (7%), using the same constraints as the initial SBRT. A key focus of this study is the detailed reporting of cumulative EQD_2_ in patients who developed RMN. The initial dose constraints were associated with favorable LC and acceptable toxicity.

## Figures and Tables

**Table 1:** Baseline patient and disease characteristics.

Variable	Number (%)
Number of lesions (patients)	61 (55)
Median Follow-up after reSBRT, months (range) *	10.3 (1–98)
Median interval between radiation courses, months (range) ^†^	10.6 (0.57–53.9)
Median Age, years (range)	65 (27.1–87.3)
Sex	
Male	38 (67)
Female	17 (33)
Karnofsky Performance Status	
≥80%	49 (89)
<80%	6 (11)
Primary diagnosis	
Lung Cancer	15 (25)
Renal Cell Carcinoma	11 (18)
Gastrointestinal	9 (15)
Thyroid	6 (10)
Sarcoma	6 (10)
Prostate	4 (7)
Head and neck	3 (5)
Urothelial	2 (3)
Neuroendocrine Tumor	2 (3)
Breast	1 (1.5)
Melanoma	1 (1.5)
Endometrial	1 (1.5)
Oligometastatic (≤5 lesions) ^‡^	
Yes	9 (16)
No	46 (84)
Spinal Instability Neoplastic Score (SINS) ^§^	
0–6	33 (54)
7–12	27 (44)
13–18	1 (2)
Bilsky grading ^§^	
0	29 (48)
1a	8 (13)
1b	8 (13)
1c	7 (11.5)
2	7 (11.5)
3	2 (3)

Abbreviations: SBRT = stereotactic body radiation therapy; SINS = spinal instability neoplastic score. * Follow-up calculated from the date of reSBRT to last clinical or imaging assessment. ^†^ Interval between radiation courses defined as the time from completion of the initial SBRT course to initiation of reSBRT. ^‡^ Oligometastatic disease defined as ≤5 metastatic lesions at the time of re-SBRT. ^§^ SINS and Bilsky grading were assessed at initial treatment. Legend: Patient-level variables include age, sex, and Karnofsky Performance Status; disease- and spine-specific characteristics were reported per lesion.

**Table 2:** Univariate and multivariate analysis for local control.

Variable	Univariate HR (95% CI)	*p*	Multivariate HR (95% CI)	*p*
LC				
Age	0.96 (0.91–1.02)	0.164	—	—
Sex (male vs female)	2.45 (0.49–12.16)	0.273	—	—
KPS	1.10 (0.98–1.22)	0.113	—	—
GI origin spine metastases	2.41 (1.11–5.23)	0.026	3.66 (1.06–12.64)	0.041
Bilsky grading	4.03 (0.47–34.51)	0.204	—	—
Spine surgery	0.86 (0.16–4.73)	0.867	—	—
Cumulative EQD_2_	0.95 (0.85–1.06)	0.351	—	—
Interval between SBRT courses	0.87 (0.75–1.02)	0.099	—	—

Abbreviations: LC = local control; HR = hazard ratio; CI = confidence interval; KPS = Karnofsky Performance Status; SBRT = stereotactic body radiation therapy; EQD_2_ = equivalent dose in 2 Gy fractions. Legend: Hazard ratios were derived from Cox proportional hazards regression models.

**Table 3:** Clinical details of the radiation myelitis or neuropathy (RMN) subgroup.

Patient	Spine Tumor Location	Initial SBRT (Gy/Fractions)	Interval to SBRT Courses (Months)	2nd SBRT (Gy/Fractions)	Systemic Therapy Within 4 Weeks	Neurologic Symptoms/Grade	MRI Findings
A *	T2-4	25/5	6	30/5	Crizotinib	right arm numbness and weakness/G2	Positive
B	L4	27/3	54	27/3	None	bilateral leg numbness/G3	Positive
C	C2	27/3	33	27/3	Pembrolizumab	bilateral hand numbness/G1	Negative
D	T12-L2 ^†^	35/5	7	30/5	Cabozantinib-Nivolumab	left foot drop/G2	Negative

Abbreviations: RMN = radiation myelitis or neuropathy; SBRT = stereotactic body radiation therapy; MRI = magnetic resonance imaging; G = grade (CTCAE v5.0). * Includes cumulative dose contribution from a prior lung radiation course. ^†^ Thoracolumbar junction case with overlapping exposure to conus medullaris, cauda equina, and nerve roots; toxicity attribution based on predominant clinical pattern. Legend: Systemic therapy refers to anticancer treatment administered within 4 weeks before or after reSBRT. Neurologic toxicity was graded according to CTCAE v5.0. MRI findings indicate radiographic evidence of radiation-related neural injury at the treated level.

**Table 4:** Dosimetric details of the non-radiation myelitis or neuropathy (non-RMN) subgroup.

SBRT-SBRT (No RMN)						
	Spinal Cord (*n* = 37)			Cauda Equina (*n* = 20)		
Metric	SBRT	reSBRT	Cumulative EQD_2_	SBRT	reSBRT	Cumulative EQD_2_
Total Rx Dose (Gy)	27.0 (24.0–35.0)	27.0 (24.0–30.0)		27.0 (27.0–27.0)	27.0 (12.0–30.0)	
Number of fractions	3 (2–5)	3 (3–5)		3 (3–3)	3 (2–5)	
Total Rx EQD_2_ (Gy_10_)	42.8 (44.0–49.6)	42.8 (36.0–40.0)	85.5 (80.0–89.6)	42.8 (42.8–42.8)	42.8 (16.0–40.0)	85.5 (58.8–82.8)
PTV D95 EQD2 (Gy_10_)	42.3 (24.3–49.6)	42.0 (15.7–44.7)	83.3 (60.0–93.8)	42.7 (39.2–44.0)	42.4 (16.0–43.3)	84.2 (58.1–86.5)
PTV D90 EQD_2_ (Gy10)	43.7 (25.1–51.9)	43.1 (23.7–45.8)	86.8 (63.7–96.9)	43.8 (41.7–45.3)	43.2 (16.5–44.2)	86.1 (59.6–88.4)
Cord/CE EQD_2_ (Gy_2_)						
D0.03 cc	19.2 (7.4–35.6)	18.6 (4.9–44.9)	27.5 (12.7–69.1)	43.8 (10.9–58.6)	34.1 (4.5–57.9)	60.6 (45.6–101.8)
D0.1 cc	16.4 (7.0–31.0)	15.7 (4.4–41.5)	23.4 (11.5–60.6)	41.5 (10.1–57.3)	31.6 (4.2–56.0)	56.2 (36.5–82.7)
Cord PRV EQD_2_ (Gy_2_)						
D0.03 cc	41.0 (15.1–86.2)	40.8 (13.3–67.0)	54.8 (32.1–121.6)	59.3 (13.3–77.0)	53.3 (9.9–75.2)	86.5 (53.5–139.6)
D0.1 cc	36.1 (13.6–73.5)	35.7 (11.7–63.8)	46.6 (28.3–110.2)	56.9 (12.4–74.2)	47.5 (8.2–72.1)	78.4 (49.8–129.4)
Thecal sac EQD_2_ (Gy_2_)						
D0.03 cc	59.9 (19.8–93.1)	55.1 (14.4–91.3)	73.8 (33.8–148.5)	72.0 (8.9–122.8)	70.7 (26.4–82.3)	92.7 (45.6–199.5)
D0.1 cc	53.0 (16.1–88.4)	49.4 (11.6–87.0)	64.4 (29.5–137.7)	67.1 (8.2–119.5)	64.5 (25.8–81.4)	83.6 (33.8–195.4)

Abbreviations: RMN = radiation myelitis or neuropathy; SBRT = stereotactic body radiation therapy; EQD_2_ = equivalent dose in 2 Gy fractions; PTV = planning target volume; PRV = planning organ-at-risk volume. Legend: All values are reported as median (range). EQD_2_ for target volumes was calculated using α/β = 10 Gy, and EQD_2_ for neural structures (spinal cord, cauda equina, cord PRV, and thecal sac) was calculated using α/β = 2 Gy. Cumulative EQD_2_ represents the sum of EQD_2_ from the initial SBRT and reSBRT courses. Spinal cord metrics apply to cervical and thoracic levels, and cauda equina metrics to lumbosacral levels. D0.03 cc and D0.1 cc represent the minimum dose to the most highly irradiated 0.03-cc and 0.1-cc volumes, respectively.

**Table 5:** Dosimetric details of the radiation myelitis or neuropathy (RMN) subgroup.

Patient	Circumferential PTV	Cumulative EQD_2_ to SC or CE		Cumulative EQD_2_ to SC PRV or CE PRV		Cumulative EQD_2_ to Thecal Sac		Cumulative EQD_2_ to Relevant Nerve Roots	
		(Gy_2_)		(Gy_2_)		(Gy_2_)		(Gy_2_)	
		D0.03 cc	D0.1 cc	D0.03 cc	D0.1 cc	D0.03 cc	D0.1 cc	D0.03 cc	D0.1 cc
A *	Yes	73.6	69.2	108.7	102.7	142.5	136.6	179.9	174.4
B	No	58.0	56.0	86.6	78.5	118.5	110.7	149.8	144.4
C	No	32.9	28.5	102.4	84.4	112.8	88.0	147.9	133.7
D	No	72.5	66.3	86.2	80.3	95.5	88.2	108.2	100.1

Abbreviations: RMN = radiation myelitis or neuropathy; EQD_2_ = equivalent dose in 2 Gy fractions; SC = spinal cord; CE = cauda equina; PRV = planning organ-at-risk volume; PTV = planning target volume. * Includes cumulative dose contribution from a prior lung radiation course. Legend: EQD_2_ values were calculated using α/β = 2 Gy for all neural structures. SC metrics apply to cervical and thoracic levels, and CE metrics to lumbosacral levels, according to anatomic applicability. D0.03 cc and D0.1 cc represent the minimum dose received by the most highly irradiated 0.03-cc and 0.1-cc volumes, respectively. Circumferential PTV indicates 360° circumferential target involvement around the neural axis.

## Data Availability

Data are available on request due to restrictions (e.g., for privacy, legal or ethical reasons).

## Abbreviations

The following abbreviations are used in this manuscript:

cEBRT	conventional external beam radiation therapy
SBRT	stereotactic body radiation therapy
SC	spinal cord
RMN	radiation-induced myelitis/neuropathy
IRB	Institutional Review Board
SINS	spinal instability neoplastic score
ESCC	epidural spinal cord compression
LC	local control
OS	overall survival
VCF	vertebral compression fracture
CE	cauda equina
GTV	gross target volume
CTV	clinical target volume
PTV	planning target volume
PRV	planning organ-at-risk volumes
SPACE	Sampling Perfection with Application-optimized Contrasts using different flip angle Evolutions
STIR	Short Tau Inversion Recovery
CSF	cerebrospinal fluid
EQD_2_	equivalent dose in 2 Gy fractions
CBCT	cone beam CT
SPINO	Spine response assessment in Neuro-Oncology
CTCAE	Common Terminology Criteria for Adverse Events
KPS	Karnofsky Performance Status
GI	gastrointestinal
